# Supplementary material for: A Novel Longitudinal Phenotype–Genotype Association Study Based on Deep Feature Extraction and Hypergraph Models for Alzheimer’s Disease
Source: Biomolecules. 2023 Apr 23;13(5):728. doi: 10.3390/biom13050728 (PMC10216733; doi:10.3390/biom13050728)
Supplement: Supplementary file 1 [file biomolecules-13-00728-s001.zip › biomolecules-2264202-supplementary.pdf]

Supplementary material

1.1 The schematic diagram of deep subspace reconstruction

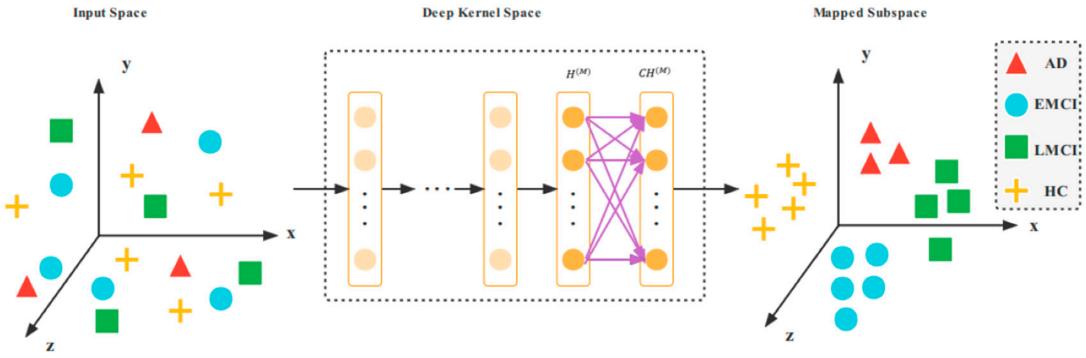

Fig. S1 The schematic diagram of deep subspace reconstruction

1.2 A hypergraph model

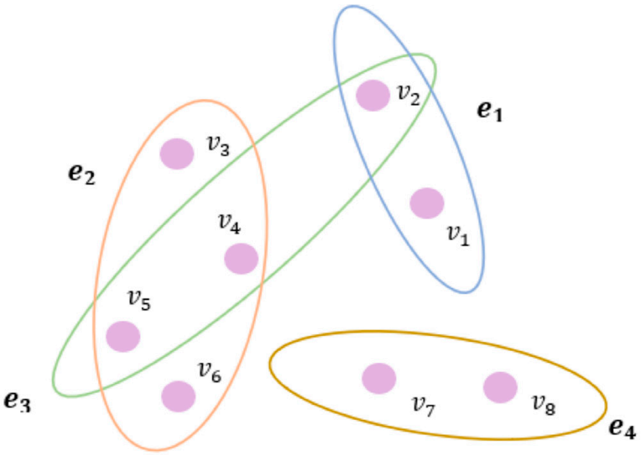

A

	e_1	e_2	e_3	e_4
v_1	1	0	0	0
v_2	1	0	1	0
v_3	0	1	0	0
v_4	0	1	1	0
v_5	0	1	1	0
v_6	0	1	0	0
v_7	0	0	0	1
v_8	0	0	0	1

B

Fig. S2 A hypergraph model（A is a hypergraph, in which each hyperedge can connect multiple nodes; B is the index matrix of A.）

1.3 The correlation coefficients of the two methods in four different periods at different noise levels

Table S1 When noise=1, the *CCCs* of the two methods in four different periods.

Algorithm	T1	T2	T3	T4
TGSCCA	0.04	0.0234	0.0504	0.0527
DS-HBTGSCCA	**0.046**	**0.0322**	**0.0571**	**0.0596**

Table S2 When noise=2, the *CCCs* of the two methods in four different periods.

Algorithm	T1	T2	T3	T4
TGSCCA	0.4765	0.4746	0.4559	**0.4616**
DS-HBTGSCCA	**0.4787**	**0.4752**	**0.4578**	0.4603

Table S3 When noise=3, the *CCCs* of the two methods in four different periods.

Algorithm	T1	T2	T3	T4
TGSCCA	0.0132	0.0129	0.0167	0.0228
DS-HBTGSCCA	**0.0342**	**0.0593**	**0.0614**	**0.0678**

Table S4 When noise=4, the *CCCs* of the two methods in four different periods.

Algorithm	T1	T2	T3	T4
TGSCCA	0.0196	0.0216	0.034	0.0197
DS-HBTGSCCA	**0.0252**	**0.0334**	**0.0392**	**0.0336**

Table S5 When noise=5, the *CCCs* of the two methods in four different periods.

Algorithm	T1	T2	T3	T4
TGSCCA	0.1701	0.1665	0.1549	0.1554
DS-HBTGSCCA	**0.1881**	**0.1834**	**0.1724**	**0.1736**

1.4 Heatmaps of high-order correlation of omics data

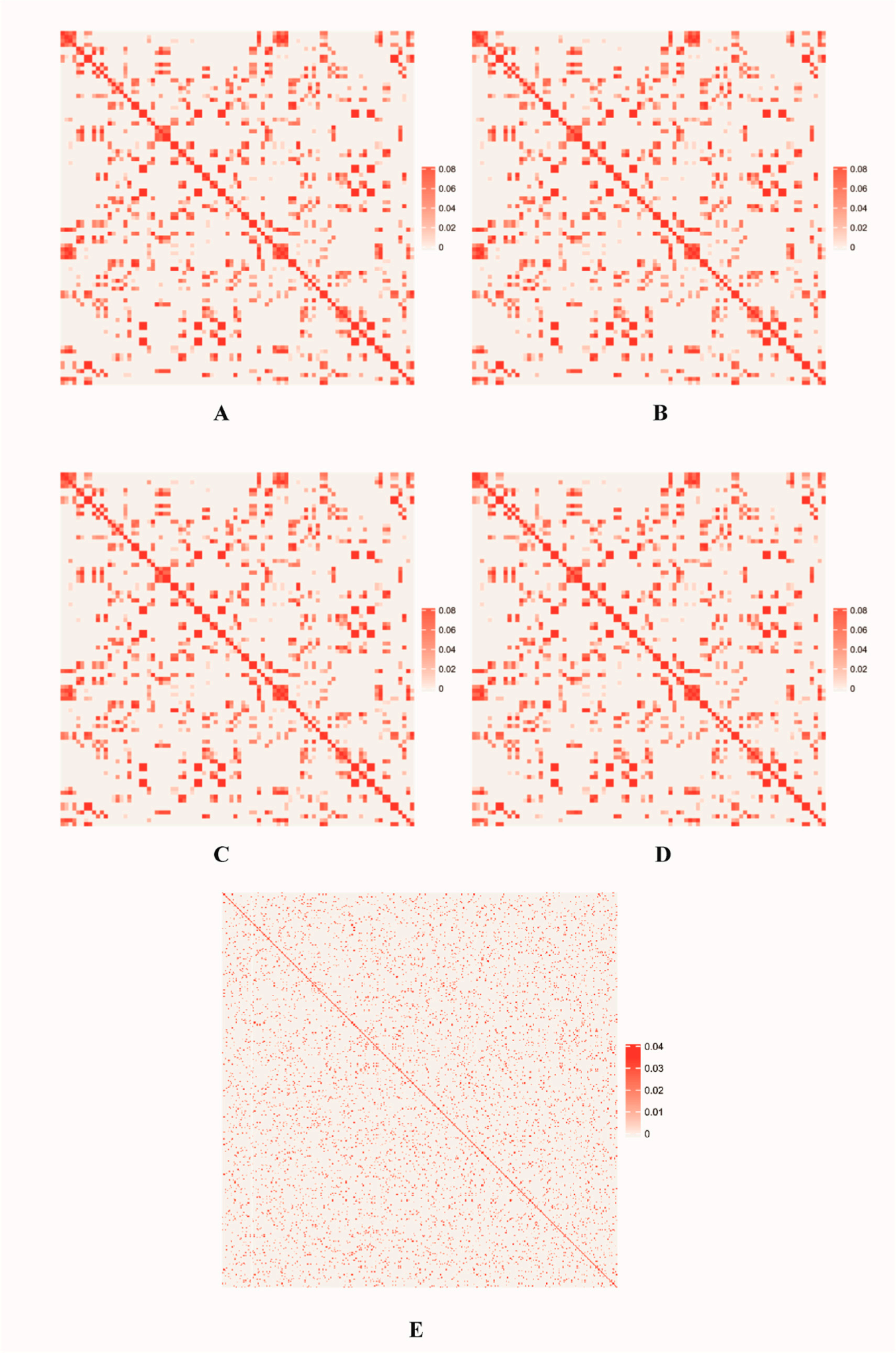

Fig. S3 Heatmaps of high-order correlation of multiomics data. A, B, C, D and E represent the heatmap of high-order correlation among T1, T2, T3, T4 and gene data, respectively, and the depth of color in the heatmap represents the high-order similarity between samples.

1.5 Evaluation of the effectiveness of depth subspace reconstruction

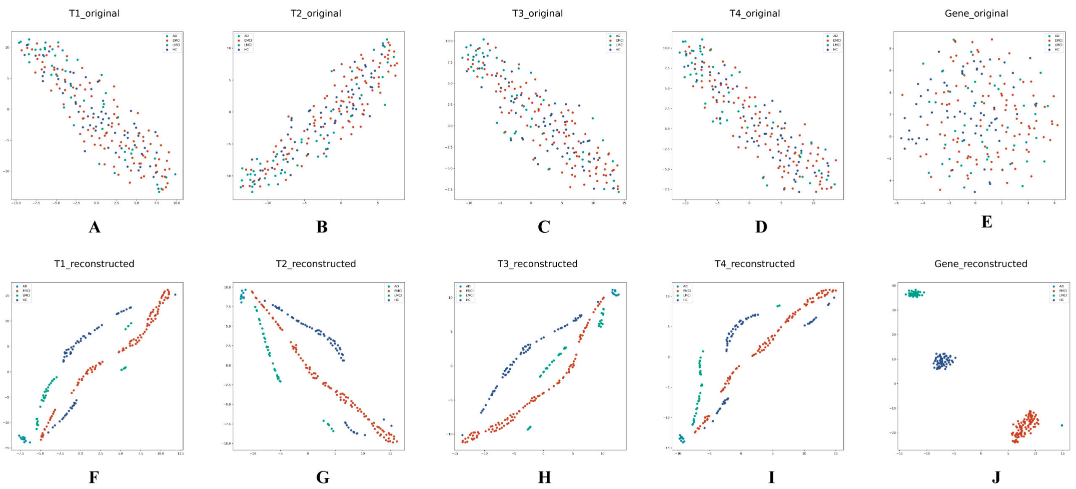

Fig. S4 The scatter plot of t-SNE dimension reduction before and after brain imaging and gene reconstruction in different periods.

Table S6 Evaluation of reconstruction effect by using silhouette_score

	T1	T2	T3	T4	Gene
Before reconstruction	-0.137	-0.106	-0.152	-0.082	-0.076
After reconstruction	-0.114	-0.117	-0.019	-0.024	1

Table S7 Evaluation of reconstruction effect by using calinski_harabasz_score

	T1	T2	T3	T4	Gene
Before reconstruction	2.758	1.702	3.568	1.457	10.414
After reconstruction	11.237	12.838	19.692	18.627	1

Table S8 Evaluation of reconstruction effect by using davies_bouldin_score

	T1	T2	T3	T4	Gene
Before reconstruction	39.766	22.903	7.127	15.43	15.23
After reconstruction	9.271	12.64	5.295	7.01	0
